# Effect of Styrene-Maleic Anhydride Copolymer on Properties of PBST/PLA Blends

**DOI:** 10.3390/polym15040952

**Published:** 2023-02-15

**Authors:** Qing Zhang, Yongguang Gao, Baojing Luo, Yan Cui, Shili Shu, Wei Chen, Lei Wang

**Affiliations:** College of Chemistry, Tangshan Normal University, Tangshan 063000, China

**Keywords:** poly(butylene succinate-butylene terephthalate), polylactic acid, styrene-maleic anhydride copolymer, blends

## Abstract

Poly(butylene succinate-butylene terephthalate) (PBST) and polylactic acid (PLA) are both biodegradable polymeric materials. PBST has good ductility but low strength, while PLA exhibits high strength but poor toughness. Based on the complementary mechanical properties of the two polymers, PBST/PLA blends were prepared by melt blending in the mixing chamber of a torque rheometer using styrene-maleic anhydride copolymer (PSMA) as a compatibilizer. The effects of different contents of PSMA on the crystalline properties, thermal properties, mechanical properties, rheological behavior, and morphology of PBST/PLA blends were investigated. The results showed that the addition of PSMA improved the compatibility between PBST and PLA. When the amount of PSMA is 3–4 wt%, the comprehensive mechanical properties of the blends are optimal, and the tensile strength was increased by 61.7% compared with the binary blend without PSMA. Additionally, rheological tests illustrated that the blends exhibited a typical shear-thinning behavior and belonged to pseudoplastic non-Newtonian fluids.

## 1. Introduction

**Scheme 1:**
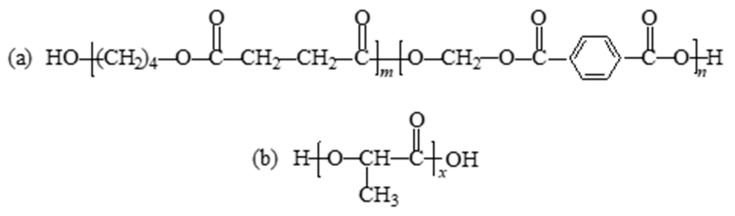
Structures of (**a**) PBST and (**b**) PLA.

Traditional petroleum-based plastics widely used in production and life, such as polypropylene, polyethylene, polyvinyl chloride, and polystyrene, have caused serious damage to the ecological environment. The application of these non-degradable polymers in packaging and other fields has been greatly impacted, and biodegradable polymers have gained more and more attention [1,2,3,4,5]. Poly(butylene succinate-butylene terephthalate) (PBST) is a biodegradable aromatic polyester, which is a copolymer of butylene succinate (BS) and butylene terephthalate (BT). The structure is shown in Scheme 1a. PBST combines the flexibility of the BS chain segment with the heat and impact resistance of the BT chain segment. It is currently one of the most promising petroleum-based biodegradable copolyesters [6,7,8]. Polylactic acid (PLA), which can be obtained from corn, wheat, sugar beet, etc., exhibits high strength, high modulus, good processing performance, good biocompatibility, but poor toughness [9,10,11,12,13]. Its structure is shown in Scheme 1b. As an important environmentally friendly bio-based polymer, PLA degrades completely to carbon dioxide and water in a composting environment [14,15]. In the actual production and research process, it is found that PBST has good ductility, but its insufficient rigidity and low strength limit its application scope [16,17], while the high strength and high modulus of PLA can compensate for these defects [18]. Based on the complementarity of the mechanical properties of PBST and PLA, it is expected to improve the comprehensive performance of the material by the melt blending of the two polymers. However, the two polymers are thermodynamically incompatible and a direct modification by melt blending will lead to lower mechanical properties [8,17]. Therefore, it is necessary to reduce the interfacial tension between the two polymers through appropriate compatibilization methods to improve the comprehensive performance of the blends.

At present, compatibilizers commonly used mainly include inorganic fillers (such as calcium carbonate, silica, nanoclay, etc.), grafted glycidyl methacrylate (g-GMA), tetrabylbutyl titanate (TBT), maleic anhydride (MAH), polyethylene glycol (PEG), epoxy resin, etc. [19,20,21,22,23]. The addition of inorganic fillers can significantly reduce production costs, but adding a large amount will deteriorate the mechanical properties of the material, whereas reactive compatibilizers can better solve such problems, among which a grafted maleic anhydride is often employed for improving the compatibility between the incompatible polyester polymers. It acts through the reaction of anhydride groups with functional groups of the polyester polymer [20,24]. For a MAH graft such as PLA-g-MAH, which is used as a compatibilizer for PLA and other polyesters, its compatibilization effect is limited due to a low content of maleic anhydride [25,26]. Styrene (St) and MAH are prone to copolymerize alternately, and the formed copolymer contains a higher content of anhydride groups. The resulting styrene-maleic anhydride copolymer (PSMA) can react with the terminal hydroxyl and carboxyl groups of PBST and PLA, which is expected to improve the compatibility between PBST and PLA and enhance the comprehensive performance of the blends.

In the present work, PSMA was prepared by the free radical copolymerization of maleic anhydride (MAH) and styrene (St). Then, PBST/PLA blends were obtained in the mixing chamber of the torque rheometer using PSMA as a compatibilizer. The structure of PMSA was determined using Fourier transform infrared (FTIR) spectroscopy. The resulting PBST/PLA blends were characterized by differential scanning calorimetry (DSC), X-ray diffraction (XRD), tensile tests, impact testing, rheological measurements, as well as field emission scanning electron microscopy (FESEM). The effects of different contents of PSMA on the crystalline properties, thermal properties, mechanical properties, rheological behavior, and morphology of PBST/PLA blends were investigated.

## 2. Materials and Methods

### 2.1. Materials

St (analytical reagent) (AR) and MAH (AR) were purchased from Fuchen Fine Chemical Company (Tianjin, China). Benzoyl peroxide (BPO) (AR), toluene (AR), 1,4-dioxane (AR), and methanol (AR) were supplied by Aladdin Industrial Co. (Beijing, China). PBST (Ecoworld) with an average molecular weight (Mw) of 128 kDa and PLA (4032D) with a Mw of 155 kDa were commercially obtained from Sinopec Yizheng Chemical Fiber Co., Ltd. (Yizheng, China) and Nature Work Company (Blair, NE, USA), respectively. Polyethylene wax (PE wax) was supplied by Qingdao Sino New Material Co., Ltd. (Qingdao, China). St was distilled under vacuum. MHA and BPO were recrystallized from chloroform and anhydrous ethanol, respectively. Other reagents were used without any further purification.

### 2.2. Preparation Procedures

#### 2.2.1. Preparation of PSMA

PSMA was synthesized by free radical copolymerization of MAH and St using BPO as an initiator according to literature [27]. Then, 98 g MA (1 mol), 104 g St (1 mol), and 2 mg BPO (1 wt%) were dissolved in 100 mL of toluene in a three-neck flask equipped with a condenser. The polymerization lasted in a 70 °C water bath under a nitrogen atmosphere, followed by a reflux with magnetic stirring for 2 h. After the reaction was completed and cooled to room temperature, the copolymer PSMA precipitated in the form of a white powder. The product was filtered off and washed with hot toluene. The copolymer was then reprecipitated from 1,4-dioxane solution into methanol and dried under vacuum at 60 °C. The molar percentage of MAH in the copolymer, calculated from the elemental analysis, was 40.6%.

#### 2.2.2. Preparation of PBST/PLA Blends

Before blending, PBST, PLA, PSMA, and PE wax were dried in a vacuum at 50 °C for 12 h to remove physisorbed surface water. The dried raw materials were weighed and premixed in a high-speed mixer for 8 min according to the formulations listed in Table 1. As an external lubricant, the amount of PE wax added was 0.6 wt%. The premixed blends were poured into the mixing chamber of a Hapro RM-200A torque rheometer (Haerbin, China) for melt blending, at the following process conditions: a rotor speed of 35 rpm, the reaction time of 10 min, and a temperature of 175 °C in the three zones. After processing in the torque rheometer, the resulting PBST/PLA blends were pelletized after cooling. Next, the blend pellets were added to the barrel of a szs-20 microinjection molder (Wuhan, China) and injected into standard splines for testing. The temperature of the mold zone and the injection zone were set to 55 and 180 °C, respectively. The injection time and the holding time were 6 s and 35 s, respectively.

### 2.3. Analyses

The structure of PSMA and PBST/PLA blends was determined by FTIR spectra, which were recorded on a Bruker VECTOR22 FT-IR apparatus (Karlsruhe, Germany), scanning 32 times from 4000 to 400 cm^−1^ room temperature.

DSC measurements were carried out on a PerkinElmer DSC 4000 instrument (Waltham, MA, USA) under a nitrogen atmosphere. The heating and cooling rates were all set to 10 °C/min. The crystallinity degree (*χ_c_*) was calculated through the following formula [28]:

$$\chi_{c}\left(\%\right)=\frac{\Delta H_{m}}{\Delta H_{m}^{0}\times \omega }\times 100\%$$

where $\Delta H_{m}$ is the melting enthalpy of the polymer component in the sample, $\Delta H_{m}^{0}$ is the melting enthalpy of 100% crystalline for PBST (121.7 J/g) [29] or PLA (93 J/g) [30], and ω is the weight fraction of PBST or PLA in the sample. $\Delta H_{m}$ was derived from the second heating DSC curve.

XRD test was conducted by a Rigaku Ultima IV X-ray diffractometer (Tokyo, Japan) with Cu Kα radiation at acceleration voltage of 40 kV and current of 30 mA.

TGA analyses were performed with a PerkinElmer TGA 4000 thermogravimetric analyzer (Waltham, MA, USA) at a heating rate of 10 °C/min from 20 to 650 °C under a nitrogen atmosphere.

Tensile properties were carried out using a CMT4202 universal testing machine (Shenzhen, China) according to ASTM D638, at a test speed of 20 mm/min. Specimens were dumbbell-shaped with dimensions of 75 mm × 4 mm × 1 mm. Izod impact properties of notched samples were determined with an XJU-22 impact tester (Suzhou, China) according to ASTM D-256 standard.

Capillary rheometry test was carried out in a RosandRH2000 double-tube capillary rheometer (Malvern, UK), using a die with a diameter of 1 mm and a length-to-diameter ratio of 16/1 under the shear rate of 100~2000 s^−1^.

Dynamic rheological tests conducted using an AR2000EX rotational rheometer (New Castle, DE, USA) equipped with a pair of parallel plates under the test temperature of 190 °C. The distance between the sample and the chassis of the rotational rheometer was 1 mm. The strain range and the angular frequency are 0.01~200% and 10 rad/s, respectively.

The morphology of the fractured surfaces of specimens was observed with a ZEISS Sigma 300 FESEM (Schnelldorf, Germany) with an accelerating voltage of 10 kV. The samples were sprayed with gold.

## 3. Results

### 3.1. FTIR Analysis of PSMA and PBST/PLA Blends

**Figure 1:**
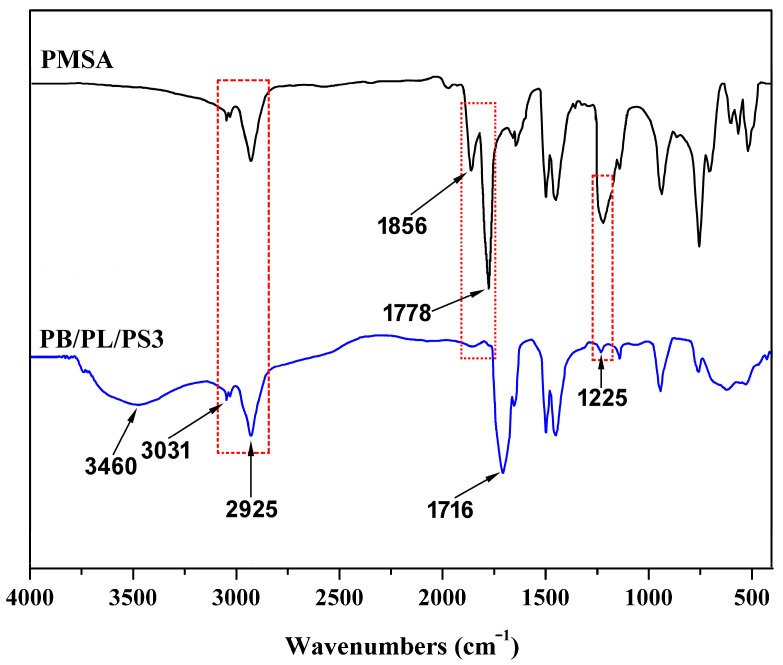
FTIR spectra of PMSA and PBST/PLA blends.

The structures of the PSMA and PBST/PLA blends obtained using PSMA as a compatibilizer were characterized using FTIR spectroscopy. As shown in Figure 1, the peaks at 2900–3100 cm^−1^ were assigned to C-H stretching vibration, among which 3031 and 2925 cm^−1^ belonged to the C-H bond on the benzene ring and the saturated C-H bond, respectively. The absorption bands which appeared at 1856 cm^−1^ and 1778 cm^−1^ were attributed to the asymmetrical and symmetrical stretching vibrations of the C=O bond in PSMA, respectively. The absorption peak at 1225 cm^−1^ was assigned to the stretching vibration of C-O-C [31] in the cyclic anhydride. These three peaks were the characteristic absorption for MHA. Bands which appeared at 1603 and 1495 cm^−1^ were assigned to stretching vibrations of the substituted benzene rings [32].

It can be seen from the spectrum of the PBST/PLA blend that the C=O stretching vibration peaks belonging to the anhydride group at 1856 and 1778 cm^−1^ and the peak at 1225 cm^−1^ assigned to the stretching vibration of C-O-C were greatly weakened and almost disappeared, indicating that the anhydride group participated in the reaction. In addition, compared with PSMA, a new characteristic absorption peak appeared at 1716 cm^−1^, which was due to the C=O stretching vibration in the newly formed ester. At the same time, the stretching vibration of -OH in the carboxylic acid generated after the esterification appeared at 3460 cm^−1^, and its shape was broad and blunt.

**Scheme 2:**
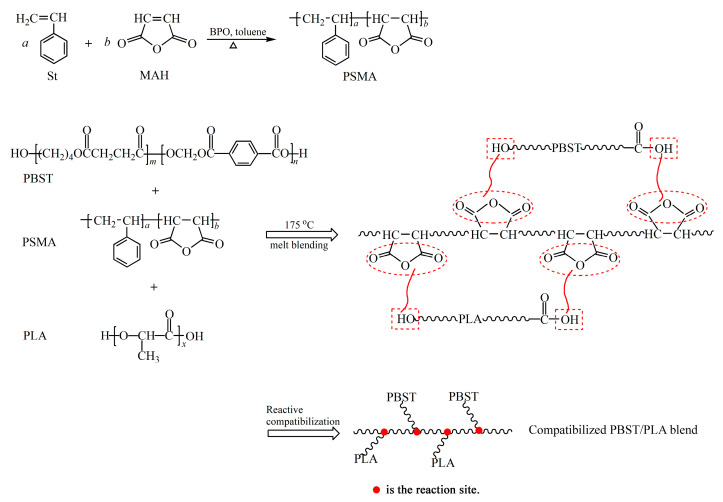
The preparation route for PSMA and PBST/PLA blend.

MAH cannot undergo homo-polymerization due to its structural symmetry and steric hindrance, but it can be copolymerized with St to obtain the copolymer PSMA. Compared with traditional grafted MAH compatibilizers, the resulting PSMA contains a higher content of anhydride groups. The terminal hydroxyl and carboxyl groups of PBST or PLA could react with the anhydride group of PSMA to increase the interfacial binding force of the two polymers, thereby improving the compatibility of PBST and PLA. Here, the OH-group of PLA did not directly bond with the carboxylic group of PBST. The esterification reaction of hydroxyl and carboxyl is a reversible equilibrium reaction with a low equilibrium constant, making the direct esterification difficult. However, in the structure of acid anhydride, the carboxyl carbon is affected by the carbonyl oxygen and another carboxyl group, making it more positively charged and easily attacked by nucleophiles. In addition, the reaction between the acid anhydride group and the hydroxyl or carboxyl group is not a reversible equilibrium reaction, which is beneficial to the reaction. The reaction process is shown in Scheme 2. (Only one possible structure of the PBST/PLA blend is given.)

### 3.2. Thermal and Crystallization Behaviors of PBST/PLA Blends

**Figure 2:**
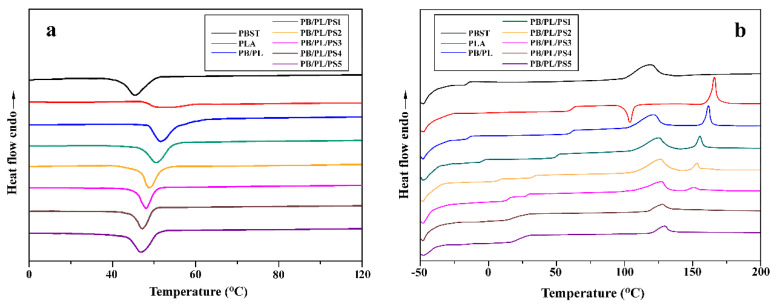
DSC curves of neat PBST, PLA, and PBST/PLA blends with various amounts of PSMA: (**a**) cooling and (**b**) the second heating.

Figure 2a,b shows the DSC cooling curves and secondary heating curves of the neat PBST, PLA, and PBST/PLA blends with different PSMA contents at a cooling rate of 10 °C/min and a heating rate of 10 °C/min, respectively. The relevant data are summarized in Table 2. It can be seen from Figure 2a and Table 2 that the crystallization temperature (*T_c_*) of the neat PBST was 45.3 °C. After adding PLA, the *T_c_* of PBST in the PB/PL binary blend shifted to a higher value, indicating that PLA has a heterogeneous nucleation effect on PBST. When the compatibilizer PSMA was added, the *T_c_* of the blends decreased with the increase in PSMA content, but it was still higher than that of the neat PBST. The reason was that the crosslinked structure generated between PBST and PLA was not conducive to the formation of crystal nuclei. Therefore, a lower temperature was required for the crystallization of the blends. However, compared with the neat PBST, the crosslinked structure still more or less hindered the molecular chain movement of PBST in the blends.

From the secondary heating curves of DSC (Figure 2b), we could see that the PB/PL binary blend without PSMA exhibited two distinctive *T_g_* values corresponding to the respective *T_g_* value of the two components, indicating the poor compatibility between PBST and PLA. As the amount of PSMA increased, the difference between the two *T_g_* values decreased gradually until 4 wt% of PSMA was added. At this point, the two values almost merged into one, which represented that the compatibility between the two components was improved greatly via adding a certain amount of PSMA. With the addition of PSMA, the melting temperature of PBST in the PBST/PLA blends presented an increasing trend, while the value of PLA in the blends decreased gradually and even disappeared when the amount of PSMA was more than 3 wt%. The cold crystallization peak (*T_cc_*) of PLA in the blends disappeared completely after the introduction of PBST. This may be because the addition of PBST reduced the mobility of the molecular chains of PLA, which hindered the transformation from the imperfect α′ to the α crystal form [33]. As shown in Table 2, when the amount of PSMA was less than 2 wt%, the crystallinity degree ($\chi_{c}$) of PBST in the PBST/PLA blends remains unchanged basically. After exceeding this percentage, the value decreased from 14.1% to 5.9% with the further increase in the amount of PSAM. Although the addition of PSMA improved the compatibility of the two polymers, the excess PSMA brought many benzene rings to the blends which were rigid groups, and caused plenty of branched polymers which were generated by the reaction of the anhydride groups with PBST and PLA. Both the rigid groups and branched polymers restricted the movement of molecular chains in the blends. Therefore, it became difficult for PBST molecules to arrange into a highly ordered lattice, thereby reducing the crystallinity. At the same time, the addition of PBST effectively weakened the friction between PLA molecular chains, which increased the crystallinity of PLA in the PB/PL binary blend. However, with the addition of PSMA, the crystallinity of PLA also decreased significantly. The reason was that a lower proportion of PLA was used as a dispersed phase and the compatibility of PBST and PLA was improved by adding PSMA, which made the PLA phase contain more PBST molecules. PLA in the blends almost lost its crystallization ability, so that the cold crystallization peak and melting peak of PLA disappeared.

**Figure 3:**
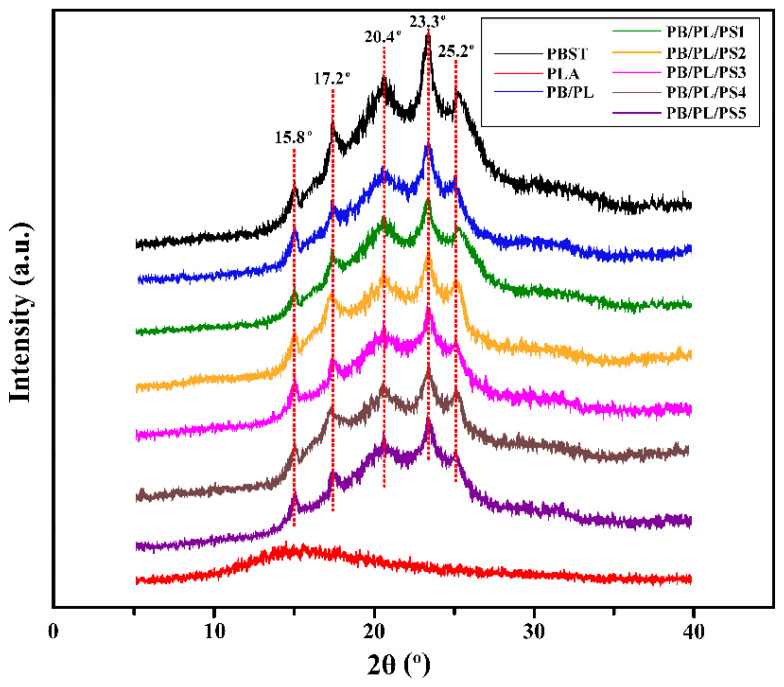
XRD spectra of neat PBST, PLA, and PBST/PLA blends with various amounts of PSMA.

The XRD patterns of the neat PBST, PLA, and PBST/PLA blends are depicted in Figure 3. PBST is a copolymer of butylene succinate (BS) and butylene terephthalate (BT). The crystal form of PBST is a mixed one of PBS and PBT, which are monoclinic and triclinic, respectively. Affected by the BT structural unit, the crystal structure of PBST is quite different from that of PBS, and it is more inclined to the crystal structure of PBT; that is, the triclinic crystal system is dominant [34]. For the neat PBST, five marked diffraction peaks were observed at 15.8°, 17.2°, 20.4°, 23.3°, and 25.2°, corresponding to (011), (010), (101), (100), and (111) crystal planes, respectively. On the contrary, PLA is predominantly amorphous, whereas only a single characteristic low intensity broad peak may be identified [35]. Compared with PBST, PBST/PLA blends exhibited almost the same diffraction peaks at the same location, indicating that PSMA had no significant effect on the crystal structure of the blends.

**Figure 4:**
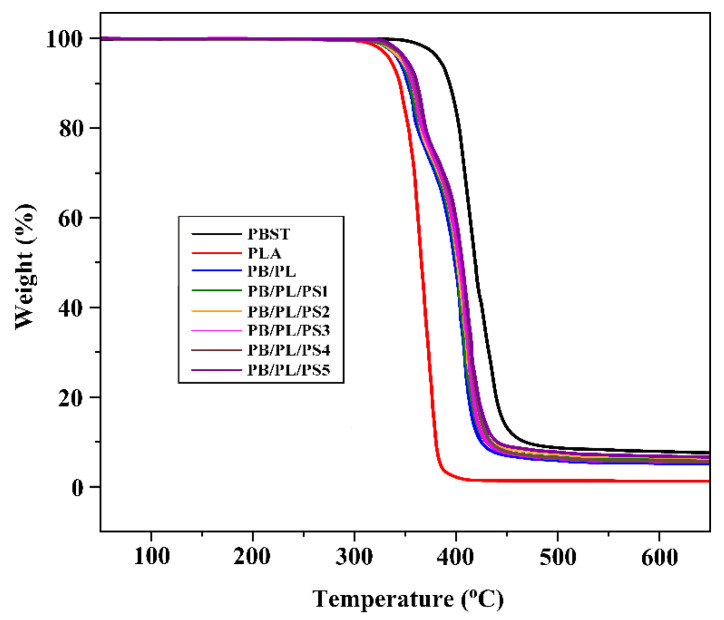
TGA curves of neat PBST, PLA, and PBST/PLA blends with various amounts of PSMA.

TGA was employed to evaluate the effect of PSMA on the thermal properties of the blends. Figure 4 gives the TGA traces of the neat PBST, neat PLA, and PBST/PLA blends with various amounts of PSMA. Thermal stabilities were determined from the temperature at 5% weight loss (T_5%_) and the temperature at 50% weight loss (T_50%_). The data are listed in Table 3. The T_5%_ and T_50%_ of the neat PLA are 331.2 and 367.4 °C, respectively, which are far lower than those of the neat PBST (379.5 and 419.2 °C, respectively). The addition of PLA reduced the thermal stability of PBST, but with the increase of PSMA content, the T_5%_ and T_50%_ of the PBST/PLA blends increased slightly again. There were two main thermal degradation stages in the TGA curves of the PBST/PLA blends. The temperature range of the first stage was from 302 °C to 375 °C, and the second stage was from 375 °C to 450 °C, which corresponded to the thermal decomposition of PLA and PBST, respectively. We could find that the compatibilizer PSMA had little effect on the thermal stability of the PBST/PLA blends, and only a slightly increase in T_5%_ and T_50%_ were observed with the increasing PSMA content. It may be that more branched copolymers were generated, which raised the degree of entanglement between molecular chains. This increased the energy required for thermal degradation, thereby improving the thermal stability of the blends.

### 3.3. Mechanical Properties of PBST/PLA Blends

**Figure 5:**
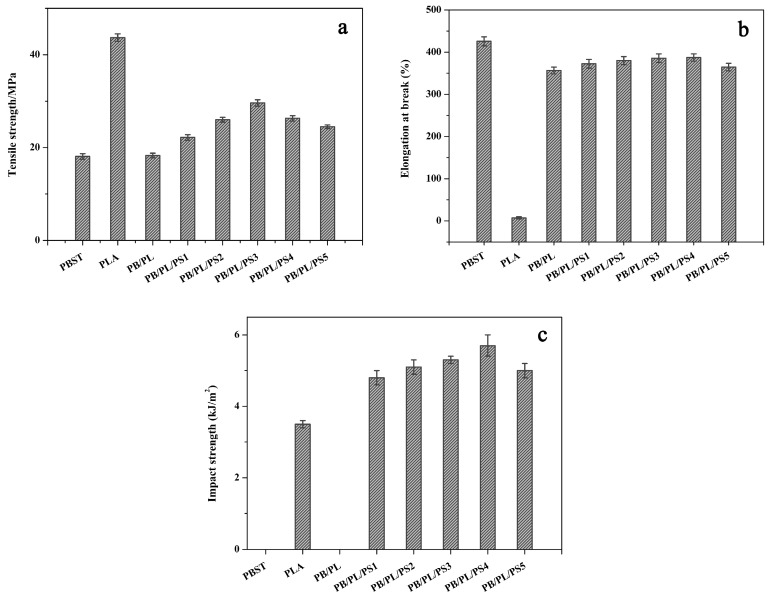
Mechanical properties of neat PBST, PLA, and PBST/PLA blends with various amounts of PSMA: (**a**) tensile strength, (**b**) elongation at break, and (**c**) impact strength.

Figure 5a–c shows the tensile strength, elongation at break, and impact strength of the neat PBST, neat PLA, and PBST/PLA blends with various amounts of PSMA, respectively. The relevant data are summarized in Table 4. The neat PBST had good ductility with up to about 425.8% of elongation at break but a low tensile strength of 18.1 MPa. In contrast, the neat PLA exhibited high rigidity with a tensile strength of 43.7 MPa, but poor impact strength and low elongation at break at the values of only 3.5 kJ/m^2^ and 7.3%, respectively. For the PB/PL binary blend, the mechanical properties were still mainly affected by the PBST component due to the low content of PLA. We can see that after adding PSMA, both the elongation at break and the tensile strength of the blends increased significantly compared with the binary blend. With the increase of PSMA content, these two values showed a trend of first increasing and then decreasing. When the PSMA content was 3 wt%, the tensile strength increased to 29.6 MPa, which was 61.7% higher than that of the binary blend without PSMA. The elongation at break reached the maximum value of 387.3% when the amount of PSMA was 4 wt%, 53 times that of neat PLA. Regarding this phenomenon, it should be noted that adding an appropriate amount of PSMA increased the compatibility between PBST and PLA, and reduced the interfacial tension of the two phases, thus improving the tensile properties of the blends to a certain extent. It was easy to see from Figure 5c and Table 4 that with the increase of PSMA content, the impact strength of the blends presented a similar change trend to that of the elongation. The impact strength rose from 4.8 to 5.7 kJ/m^2^ when the PSMA content increased from 1 to 4 wt%. However, with the further increase of the PSMA content in the blends, the value declined. This was also because the anhydride group of PSMA reacted with the carboxyl groups or hydroxyl groups at the end of the PBST and PLA chain, thereby enhancing interfacial adhesion of the two polymers. However, with the further increase of PSMA, excess anhydride groups easily led to cross-linking and then hindered the movement of chain segments, resulting in a decrease in the impact strength.

### 3.4. Rheological Behavior of PBST/PLA Blends

#### 3.4.1. Capillary Rheometry

**Figure 6:**
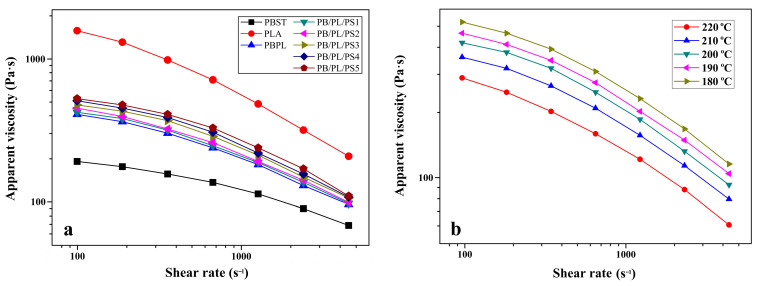
Variation of apparent viscosity as a function of shear rate: (**a**) neat PBST, PLA, and PBST/PLA blends with various amounts of PSMA, and (**b**) PB/PL/PS3 under different temperatures.

The shear-flow behavior of the neat PBST, PLA, and PBST/PLA blends with various amounts of PSMA was analyzed. The variation of apparent viscosity (*η*_a_) as a function of the shear rate ($\dot{\gamma }$) under 200 °C was presented in Figure 6a. It was seen that the *η*_a_ of the neat PBST, neat PLA, and PBST/PLA blends all decreased with the increase of $\dot{\gamma }$ and exhibited a typical shear-thinning behavior of polymer materials, indicating that these polymers were pseudoplastic non-Newtonian fluids. With the increase of PSMA content, the *η*_a_ of the blends increased. This may be due to the formation of more copolymers between PBST and PLA through anhydride groups, which increased the entanglement between molecular chains. Moreover, the benzene rings on the PSMA structure also hindered the movement of molecular chains to a certain extent, thereby manifesting as an increase in viscosity macroscopically. The effect of temperature on the *η*_a_ of the PBST/PLA blends with 3 wt% PSMA was investigated, as shown in Figure 6b. At the same shear rate, the *η*_a_ of the blend was highly sensitive to the temperature, showing a decreasing trend with the increasing temperature, which was attributed to the fact that the increasing temperature weakened the interaction between molecular chains, made the thermal motion of molecular chains more intense, reduced the entanglement between molecular chains, and accordingly accelerated the fluidity of the melt [36]. Another reason was that the free volume of polymer materials was positively correlated with the temperature; that is, the higher the temperature, the larger the free volume. The high degree of freedom for molecular chain movement made it easier to be oriented under the same $\dot{\gamma }$. Therefore, from a macroscopic point of view, it was shown that the *η*_a_ gradually decreased with the increase in the temperature.

**Figure 7:**
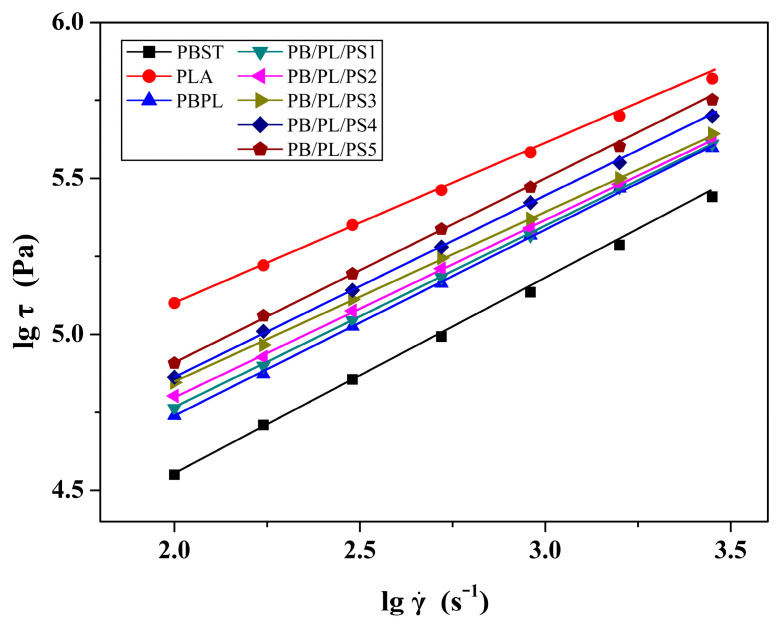
Variation of lg*σ* as a function of lg$\dot{\gamma }$ of samples under 190 °C.

The relationship between the shear stress (*σ*) and shear rate ($\dot{\gamma }$) of the neat PBST, PLA, and PBST/PLA blends with various amounts of PSMA (T = 190 °C) was presented in Figure 7. The value of the shear stress exhibited an upward trend as the shear rate increased. In addition, at the same shear rate, the shear stress also increased with the increase of PSMA content. Meanwhile, there was an obvious linear relationship between the shear stress and the shear rate. The non-Newtonian index (*n*) of the melt could be obtained according to the formula $n=\frac{dlg\sigma }{dlg\dot{\gamma }},$ that is, the slope of the curve. The non-Newtonian index was employed to indicate the degree to which the melt deviated from the Newtonian fluid. The smaller the value, the more dramatically the apparent viscosity decreased with the increasing shear rate. The non-Newtonian indices of the PBST/PLA blends with various amounts of PSMA under different temperatures were listed in Table 5. It was found that with the increasing PSMA content at the same temperature, the non-Newtonian index first decreased and then increased with the increase of PSMA content. When the PSMA content in the blends increased to 3 wt%, the non-Newtonian index decreased to the minimum value. This indicated that the non-Newtonian property of the blend PB/PL/PS3 was the strongest, and its melt viscosity was most sensitive to the shear rate. The enhanced pseudoplasticity of the melt implied a more pronounced reduction in viscosity as the shear rate increased. After the amount of PSMA exceeded 3 wt%, the non-Newtonian index of the blend increased in turn. This was because the addition of excess PSMA increased the macromolecular entanglement points of the blends, weakened the fluidity of the melt, and thus increased the viscosity. From the influence of temperature on the non-Newtonian index, we can see that for a certain blend, the index increased with the increasing temperature, which meant that the non-Newtonian property of the melt was weakened. As the temperature rose, the energy supplied to the polymer molecular chains increased. The intense movement of molecular chains weakened the entanglement between macromolecules, making the apparent viscosity less dependent on the shear rate.

#### 3.4.2. Dynamic Rheological Properties

**Figure 8:**
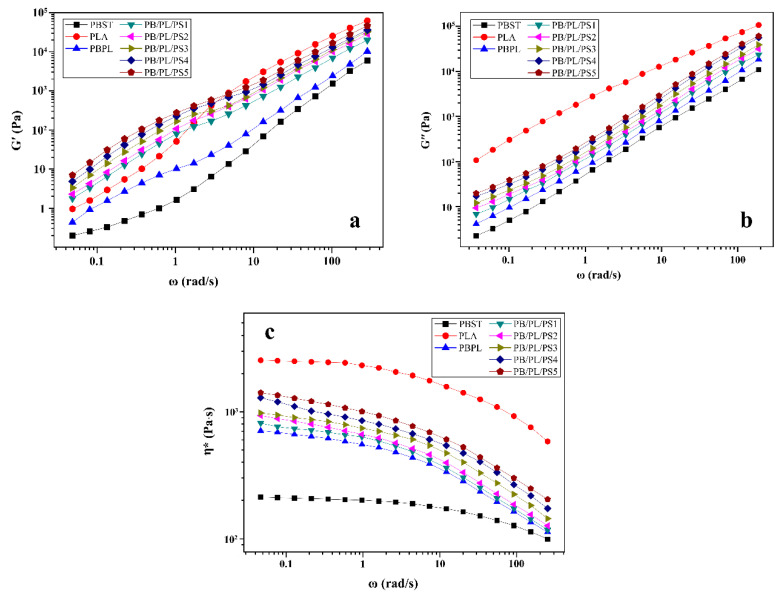
Curves of dynamic frequency sweep for neat PBST, PLA, and PBST/PLA blends with various amounts of PSMA: (**a**) G′, (**b**) G″, and (**c**) η*.

The storage modulus (G′) and loss modulus (G″) reflect the elasticity and viscosity of the material in the molten state, respectively. Figure 8 displayed the curves of the dynamic frequency sweep for the neat PBST, PLA, and PBST/PLA blends with various amounts of PSMA. It was observed from Figure 8a and b, with the increase of angular frequency (*ω*), both the G′ and G″ of the samples showed an increasing trend. It was worth noting that in the low frequency region, the G′ of the blends was greater than that of the neat PBST and PLA, while the G′ of the blends was located between the two polymers in the high frequency region. For the blends, the enhanced elasticity in the low frequency region could be due to the shape relaxation of the dispersed phase under the oscillatory shear flow, which was typical for a blend of two Maxwellian fluids with different interfacial tension [16]. After adding PSMA, the G′ and G″ of the PBST/PLA blends were significantly higher than those of the binary blend without PSMA. In addition, as the amount of PSMA increased, the values increased. This demonstrated that the addition of PSMA improved the interfacial cohesion between the two polymers and increased the entanglement ability between the PBST and PLA molecular chains. Therefore, in order to carry out the relative movement between molecular chains, it was necessary to overcome the greater slip resistance and lose more energy [37], resulting in an increase in the G′ and G″.

The relationship between the complex viscosity (η*) and angular frequency (*ω*) of all the samples was presented in Figure 8c. The complex viscosity of the neat PBST and PLA remained basically unchanged in the low frequency region, while decreased with the increase of angular frequency in the high frequency region, showing an obvious Newtonian plateau at a low angular frequency and a slight shear-thinning behavior in the high frequency region, respectively. For the PBST/PLA blends, the η* laid between the neat PBST and neat PLA in the entire frequency, and increased with increasing amount of PSMA at the same angular frequency. Moreover, the blends exhibited a pronounced shear-thinning behavior, and this behavior became stronger with increasing angular frequency. It may be that with the increase in the angular frequency, the molecular chains of the blends were disentangled and the elastic response of the molecular chains was improved in a short time [38].

### 3.5. SEM Analysis

**Figure 9:**
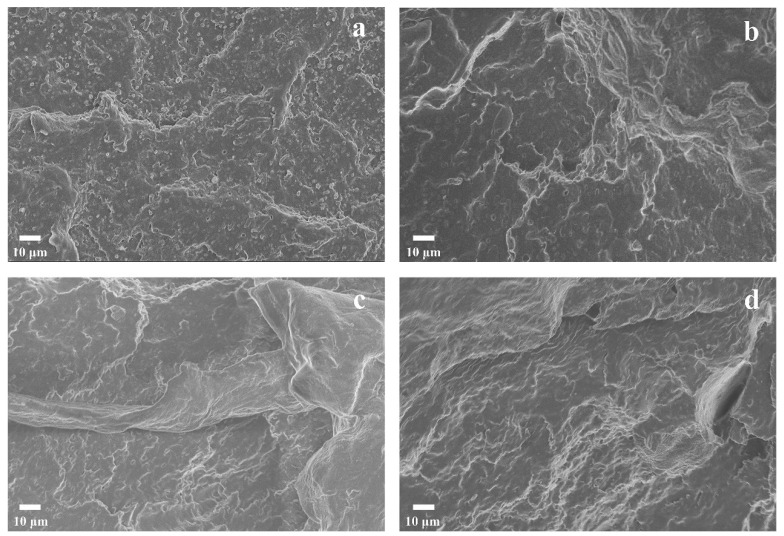
SEM micrographs of impact-fractured surfaces of PBST/PLA blends with various amounts of PSMA: (**a**) PB/PL, (**b**) PB/PL/PS1, (**c**) PB/PL/PS2, and (**d**) PB/PL/PS3 blends.

SEM characterization was performed on the impact-fractured surfaces of the PBST/PLA blends with various amounts of PSMA, as shown in Figure 9. For the PB/PL binary blend without PSMA (Figure 9a), the dispersed phase (spherical PLA particles) could be distinguished from the PBST matrix, and there was a clear boundary between the dispersed-phase and the continuous-phase PBST matrix, presenting a typical “sea–island” structure. In addition, there were obvious holes on the fracture surface. These indicated that the bonding force of the two phases was poor; some dispersed phase particles of the blends fell off from the matrix PBST under the external force. Simply mixing the two polymers could lead to poor compatibility, weak interfacial adhesion, and a distinct phase interface between PBST and PLA. With the increase of PSMA content (Figure 9b,c), the interface between the two phases began to blur, and the particles of the dispersed phase were gradually wrapped by the continuous phase. Moreover, the fracture surface became smoother, and the size of the dispersed phase became significantly smaller, until the amount of PSMA reached 3 wt% or more, and the fracture surface of the blends presented a “sea–sea” structure. This phenomenon may be due to the fact that the copolymers were formed at the interface of the two phases via the reaction of anhydride groups with PBST and PLA, which hindered the aggregation of PLA in the continuous phase to some extent. It demonstrated that the addition of an appropriate amount of PMAS effectively increased the interfacial bonding force and improved the compatibility of PBST and PLA.

## 4. Conclusions

PBST/PLA blends were prepared by melt blending using PSMA as a compatibilizer in the present work. The effects of different contents of PSMA on the thermal properties, crystalline properties, mechanical properties, rheological behaviors, and morphology of the PBST/PLA blends were investigated. SEM results showed that PBST was immiscible with PLA, and PLA dispersed as spherical particles in the PBST matrix, while the addition of PSMA effectively improved the compatibility between the two polymers. The compatibility was also verified by DSC tests. With the increase in the amount of PSMA, the difference in *T_g_* between PBST and PLA decreased until they almost merged into one. Both the crystallization temperature and crystallinity of PBST in the blends were reduced after the addition of PSMA, but it had no significant effect on the crystal structure of the blends. In addition, when the amount of PSMA is 3–4 wt%, the comprehensive mechanical properties of the blends are optimal, and the tensile strength was increased by 61.7% compared with the binary blend without PSMA. Rheological tests illustrated that the blends exhibited a typical shear-thinning behavior and belonged to pseudoplastic non-Newtonian fluids.

## Figures and Tables

**Table 1:** Formulations investigated in the present study.

Samples	PBST (wt%)	PLA (wt%)	PSMA (wt%)
PBST	100	0	0
PLA	0	100	0
PB/PL	70	30	0
PB/PL/PS1	70	30	1
PB/PL/PS2	70	30	2
PB/PL/PS3	70	30	3
PB/PL/PS4	70	30	4
PB/PL/PS5	70	30	5

**Table 2:** DSC data for PBST/PLA blends.

Samples	*T_c_* (°C)	*T_g_* (°C)	*T_m_* (°C)	$\chi_{c}\;(\%)$
PBST	45.3	−16.2	119.0	14.5
PLA	-	61.7	166.3	10.8
PB/PL	51.7	−14.5/60.4	122.1/161.6	14.2/19.3
PB/PL/PS1	50.8	−4.3/50.8	125.9/155.6	14.3/14.8
PB/PL/PS2	48.7	7.8/33.4	127.3/153.5	14.1/8.5
PB/PL/PS3	48.1	14.9/28.5	127.7/151.2	10.5/7.2
PB/PL/PS4	47.3	20.8	128.1	6.3
PB/PL/PS5	46.9	22.3	129.6	5.9

**Table 3:** TGA data for neat PBST, PLA, and PBST/PLA blends.

Samples	T_5%_ (°C)	T_50%_ (°C)
PBST	379.5	419.2
PLA	331.2	367.4
PB/PL	338.0	398.2
PB/PL/PS1	340.6	400.9
PB/PL/PS2	341.3	401.7
PB/PL/PS3	344.5	403.1
PB/PL/PS4	344.9	403.6
PB/PL/PS5	346.7	406.1

**Table 4:** Data for the mechanical properties of PBST/PLA blends.

Samples	Tensile Strength (MPa)	Elongation at Break (%)	Impact Strength (kJ/m^2^)
PBST	18.1 ± 0.6	425.8 ± 10.7	No fracture
PLA	43.7 ± 0.8	7.3 ± 2.5	3.5 ± 0.1
PB/PL	18.3 ± 0.5	356.6 ± 8.2	No fracture
PB/PL/PS1	22.2 ± 0.6	372.5 ± 10.3	4.8 ± 0.2
PB/PL/PS2	26.0 ± 0.5	380.1 ± 9.6	5.1 ± 0.2
PB/PL/PS3	29.6 ± 0.7	385.6 ± 10.1	5.3 ± 0.1
PB/PL/PS4	26.3 ± 0.6	387.3 ± 8.5	5.7 ± 0.3
PB/PL/PS5	24.5 ± 0.4	364.5 ± 9.0	5.0 ± 0.2

**Table 5:** Data for the non-Newtonian indices of PBST/PLA blends with various amounts of PSMA under different temperatures.

Samples	Non-Newtonian Index (*n*) ^a^				
	180 °C	190 °C	200 °C	210 °C	220 °C
PB/PL	0.572 ± 0.012	0.586 ± 0.039	0.601 ± 0.031	0.615 ± 0.043	0.630 ± 0.025
PB/PL/PS1	0.562 ± 0.025	0.574 ± 0.031	0.587 ± 0.022	0.600 ± 0.036	0.612 ± 0.017
PB/PL/PS2	0.544 ± 0.017	0.556 ± 0.026	0.570 ± 0.028	0.584 ± 0.030	0.599 ± 0.026
PB/PL/PS3	0.526 ± 0.034	0.439 ± 0.018	0.550 ± 0.026	0.562 ± 0.029	0.575 ± 0.023
PB/PL/PS4	0.545 ± 0.019	0.560 ± 0.023	0.573 ± 0.030	0.587 ± 0.016	0.601 ± 0.038
PB/PL/PS5	0.566 ± 0.022	0.578 ± 0.027	0.591 ± 0.036	0.605 ± 0.041	0.619 ± 0.020

^a^*n* was taken as the arithmetic mean in the range of low shear rates.

## Data Availability

Not applicable.
